# Positive ROS (Reactive Oxygen Species) Modulator Engineered Device Support Skin Treatment in Locally Advanced Breast Cancer (LABC) Enhancing Patient Quality of Life

**DOI:** 10.3390/jcm11010126

**Published:** 2021-12-27

**Authors:** Donato Casella, Paolo Palumbo, Sara Sandroni, Claudio Caponi, Francesca Littori, Francesca Capuano, Luca Grimaldi, Marco Marcasciano, Roberto Cuomo

**Affiliations:** 1 Oncological Breast Surgery, Department of Surgery, Santa Maria Alle Scotte Hospital, 53100 Siena, Italy; donato.casella@ao-siena.toscana.it (D.C.); dott.marcomarcasciano@gmail.com (M.M.); 2 Casa di cura Villa Fiorita-Prato, 59100 Prato, Italy; 3 Senior Nurse Manager Network Wound Care Azienda USL Toscana Sud Est, 52100 Arezzo, Italy; sara.sandroni@uslsudest.toscana.it; 4 Breast Unit, Oncology Department, Empoli Hospital, 50053 Empoli, Italy; claudio.caponi@uslcentro.toscana.it (C.C.); francesca.littori@uslcentro.toscana.it (F.L.); 5 Oncology Department, 59100 Prato, Italy; francesca.capuano@uslcentro.toscana.it; 6 Plastic and Reconstructive Surgery, Department of Medicine, Surgery and Neuroscience, University of Siena, 53100 Siena, Italy; luca.grimaldi@unisi.it (L.G.); robertocuomo@outlook.com (R.C.)

**Keywords:** ROS, breast cancer, polyester scaffold, pain, exudate, wounds

## Abstract

The development of research in genetic and biochemical fields has made it possible to investigate certain metabolic aspects of the microenvironment of chronic skin lesions, including altered cell signalling, highlighting its importance in determining the blockage of repair processes. The purpose of this prospective observational study is to evaluate the efficacy of a medical device consisting of a polyester scaffold enriched with an oleic matrix with controlled release of ROS in the management of LABC skin lesions. During the period from October 2018 to March 2020, 20 patients with locally advanced breast cancer were enrolled and ten were treated with the devices abovementioned. After 30 days of treatment all patients treated reported a general improvement in local conditions with reduction in ulceration area, exudate and odour. The results suggest that the application of these devices even in particular conditions (healthy and neoplastic tissue) does not lead to the onset of negative effects due to the release of ROS, though their role in tissue repair requires further study to fully understand their potential and increase the fields of application of the device by exploiting its modulation capabilities.

## 1. Introduction

The development of research in the genetic and biochemical fields impulse the investigation of certain metabolic aspects of the microenvironment of chronic skin lesions, including altered cell signalling, highlighting its importance in determining the blockage of repair processes. Among the primary causes of repair failure, inflammation appears to be the main culprit and a key role in its maintenance has been identified in the redox system.

The redox system is capable of producing reactive oxygen species (ROS) whose concentration at the cellular level dynamically influences metabolic and environmental signaling responses [1].

By exploiting the ability to store oxygen and ozone in an olive oil matrix, a gel has been developed that can modulate the response of the injured tissue. This gel was then incorporated into a polyester scaffold where the release of the substance occurs gradually and can be packaged according to the treatment area (e.g., as a breast cup or bra cup) [2].

The combination is a class IIb Medical Device, based on oxygen-enriched oil releasing reactive oxygen species (ROS), supported on a polyurethane sponge with polyester fabric external layers. The support ensures a proper contact between the oxygen-enriched oil and the wound, and healing processes are positively influenced by ensuring a moist environment, promoting oxygenating tissue, stimulating a revascularized granulation bed and consequent re-epithelialization, and preventing secondary infection.

The device is intended to treat the following breast lesions and scars:

- Surgical wounds following total or partial oncological and reconstructive surgery;
- Surgical wounds following aesthetic surgery;
- Hypertrophic scars;
- Burns;
- Superficial wounds (including fissures);
- Traumatic wounds (such as abrasions and lacerations);
- Ulcers or open surgical wounds;
- Radiotherapy ulcers and wound.

As a consequence of ROS release, oxygen-enriched oil already is recognized in multiple fields as an adjuvant to suppress bacterial colonization and pathogenesis proliferation, as well as a debriding agent promoting a microenvironment capable of stimulating angiogenesis [2,3].

Functioning as secondary messengers to immunocytes and nonlymphoid cells involved in tissue repair, ROS usually recruit immune factors and encourage angiogenesis at wound sites [4]. Moreover, in the case of its exogenous application to a wound bed, antimicrobial activity is augmented and the enhancement of tissue oxygenation leads to local immune stimulation and wound closure [2]. The concept of reactive species modulation (ROS) involves the release of constant quantities of reactive oxygen species for prolonged periods at low doses, differing substantially from other devices that incorporate large quantities of ozone, which, being highly unstable, tends to be immediately broken down into reactives and to exhaust its action in a short time.

Locally advanced breast cancer (LABC) is now an uncommon condition due to screening and surveillance programmes for breast lesions, and the treatment approach has also changed; nowadays, surgery is rarely an option since neoadjuvant therapy (chemotherapy and radiotherapy) represents the first-line therapy and can seldom act as a ‘bridge’ to surgery to remove the breast mass [5]. During this period, however, the frequently secreting, malodorous and painful lesions are negative factors that severely affect patient quality of life and require daily medications to clean the lesions, though they have little effect on inflammation and pain. In the case of large and multiple lesions, the malodorous exudate and the pain have been found to induce depression and reduce social interaction [6].

Ulcerated stage III and stage IV breast cancer remains difficult to treat and survival in this population is driven by local and distant progression of the neoplastic disease. Consequently, systemic therapy has remained the cornerstone of treatment and the role of local therapy remains controversial [7,8].

The purpose of this prospective observational study is to evaluate the efficacy of a medical device consisting of a polyester scaffold enriched with an oleic matrix with controlled release of ROS in the management of LABC skin lesions.

## 2. Materials and Methods

This study was approved by the Ethics Committee and was conducted in accordance with the Helsinki Declaration of 1964 (revised in 2008) and all patients gave their informed consent.

This study is a prospective observational study and was conducted between October 2018 and March 2020.

Patients were recruited by applying the following inclusion criteria:

− Age up to 75 years;
− Presence of locally advanced/metastatic breast cancer;
− Presence of ulcerated/esophitic lesions with secretions.

Each patient underwent an anamnesis and clinical evaluation, and a thorough staging of the disease was performed by mammography, breast ultrasound with biopsy for micro-histological examination, bilateral breast MRI, bone scintigraphy and total body CT/PET.

All cases were evaluated by a multidisciplinary team of specialists who, after confirming that surgery was not feasible, opted for chemotherapy treatment combined with local medication.

First-line therapy was medical treatment for all patients. Luminal-a patients (11 cases) were treated with hormonal therapy, scheduled for at least 6 months. Luminal-b1 (ki-67 > 20% and Her-2 negative, 3 cases) and triple negative patients (3 cases) were treated with chemotherapy (4 cycles of epirubicin + cyclophosphamide every 21 days followed by 12 weekly paclitaxel cycles). Luminal-b2 (her-2 positive, 1 case) and her-2 positive patients (2 cases) were treated with chemotherapy as well (4 cycles of epirubicin + cyclophosphamide every 21 days followed by 12 weekly paclitaxel + trastuzumab cycles).

A total of twenty (20) patients were included and divided into two different groups (Group A; Group B) of 10 patients each. Two local therapy protocols were administered to each group.

The criterion adopted for the selection of the patients was the sequential criterion in order to guarantee the randomness of the samples, since there was no way of knowing which patients would present over time with the inclusion criteria described above.

All patients were given the medical treatment prescribed before the start of the study, including painkillers and anti-emetics if needed.

In group A, the protocol consisted of the following steps:

1. Wound cleansing with 0.9% NaCl solution;
2. Application of oxygen-enriched oleic matrix gel to the wound surface (including peri-injury skin and surrounding intact skin up to 2 cm);
3. Application of a preformed cup device in polyester with polyurethane soaked in an oxygen-enriched oleic matrix; or
4. In the case of excessively large lesions, additional application of gauze soaked in an oxygen-enriched gel matrix.

In group B, the protocol consisted of the following steps:

1. Cleaning with sodium hypochlorite solution;
2. Washing with saline solution;
3. Application of silver sulfadiazine ointment on gauze soaked in neutral fat.

In both groups the dressing was left in place for 48 h and then replaced twice a week until day 30.

Silver sulfadiazine was chosen because in our setting it is considered “standard of care”. It is usually present in all outpatient rooms, especially in outpatient rooms for palliative care which does not include advanced dressings, and it is used often for the management of these kind of wounds due its ability to reduce wound contamination.

Lesion assessment was performed using a score (attributing from 0 to 5 points—e.g., 0 = absence/5 = maximum amount of exudate) based on the TIME [9] criteria (stands for Tissue, Infection, Moisture and Edge) based on:

- Size of the lesion;
- Amount of exudate;
- Presence of infection;
- Presence of necrosis.

Photographs were taken at the beginning of local therapy and at dressing renewal until day 30.

Quality of life was assessed by administering the Wound-QoL 6 questionnaire before and after the treatment period. The following parameters were investigated in the questionnaire:

- Influence of injury on interpersonal relationships;
- Influence of the wound on rest;
- Emission of foul odour from the wound;
- Production of irritating secretions from the wound;
- Influence of the wound on the state of mind.

(The above parameters were also scored from 1 to 5, where 0 corresponded to no symptoms and 5 to maximum symptomatic influence.)

Pain perception was assessed according to the Pain Intensity Scale before and after treatment, describing pain intensity in a range from 0–10 (0 = no pain, 10 = maximum pain) [10].

## 3. Results

During the period from October 2018 to March 2020, 20 patients with LABC aged between 58 and 75 years (mean 69.3 years) and weighing between 58 and 87 kg (mean 67.6 kg) were included in the study. All patients presented with unilateral localization of the primary neoplasm with exophytic lesions in ulcerative phase. In the peri-lesional site there was erythema involving the whole mammary region and the skin of the ipsilateral hemithorax. In the context of the ulcerative lesions there were necrotic areas covered with eschar, alternating with secreting and malodorous tissue.

### 3.1. Impact on Pain

All patients reported pain. After 30 days of treatment, all patients in group A reported a general improvement in local conditions with a reduction in ulceration area, exudate and odour. The pain disappeared in one patient, was relieved by reducing the intake of painkillers in six, and continued analgesia at the initial dosage was necessary in the remaining three. In group B, the reduction in lesion area was approximately 50% less than in group A.

All patients reported an improvement in pain associated with the injury. According to the Pain Intensity Scale, the two groups had almost identical values at the start of treatment, which were significantly reduced in group A compared with group B.

**Scheme 1:**
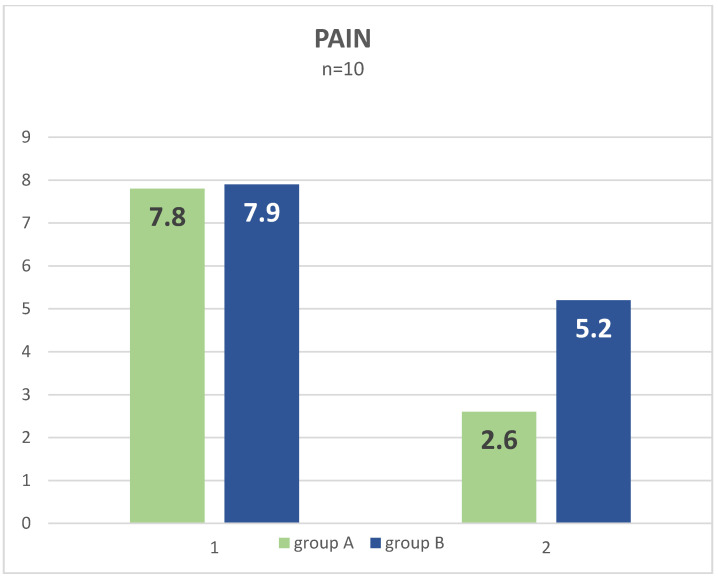
Pain.

**Scheme 2:**
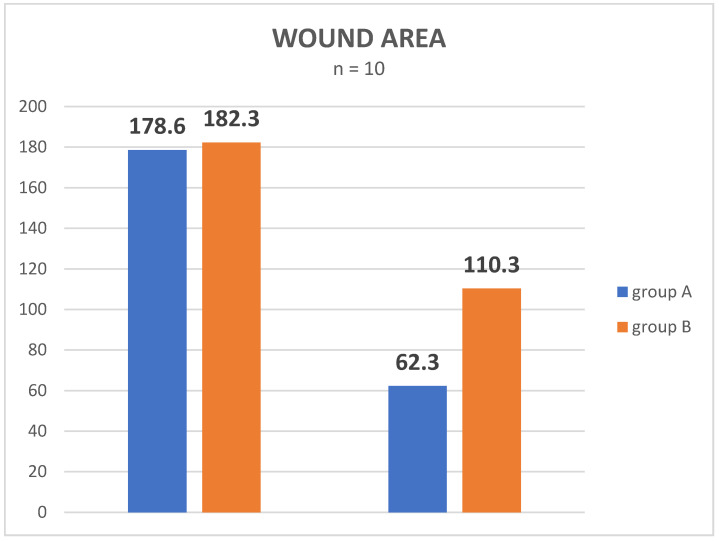
Wound area.

**Scheme 3:**
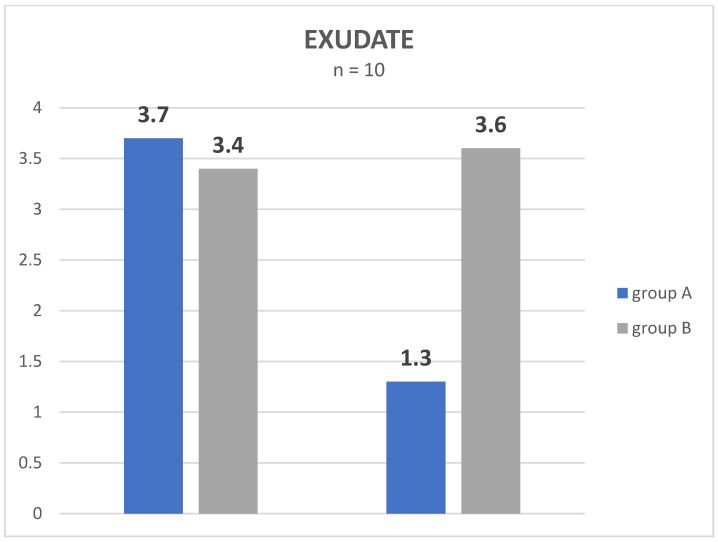
Exudate.

**Scheme 4:**
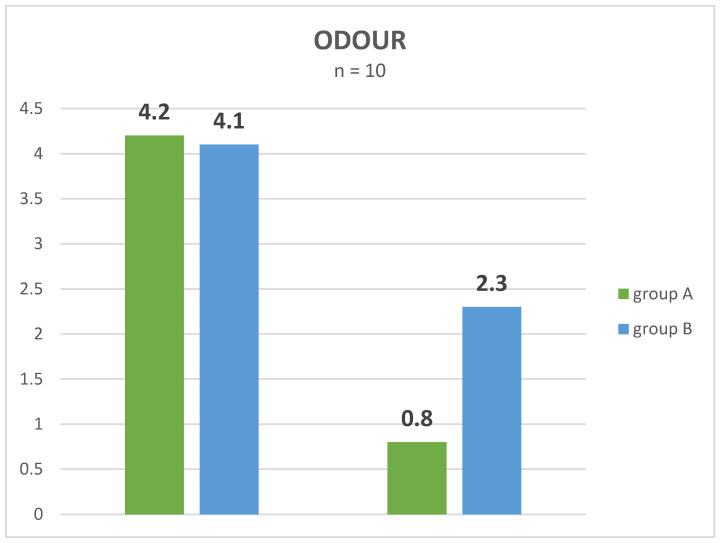
Odour.

**Scheme 5:**
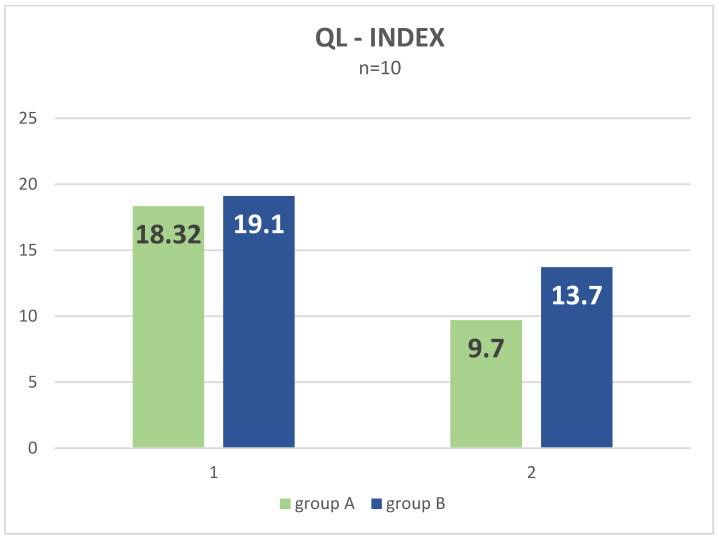
QL-index.

The reduction in pain was associated with a simultaneous reduction in exudate and odour, which was observed mainly in group A (see Scheme 1, Scheme 2, Scheme 3, Scheme 4 and Scheme 5).

The reduction in pain, exudate, odour, wound area and QL index was observed mainly in group A.

### 3.2. Necrosis

**Figure 1:**
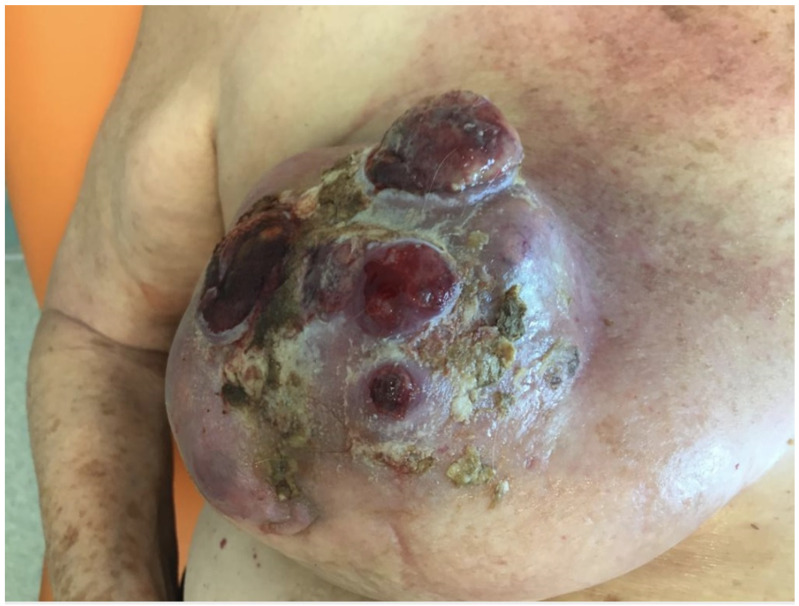
Necrotic area before the application.

**Figure 2:**
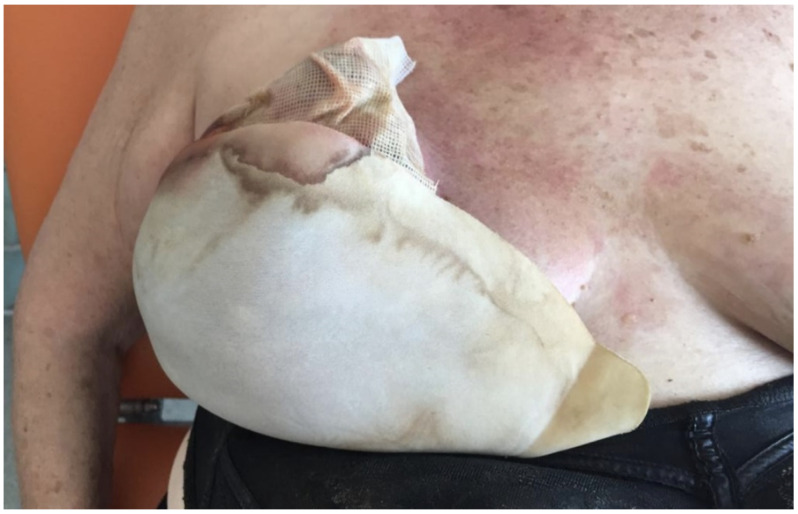
The device is applied to cover all the breast.

**Figure 3:**
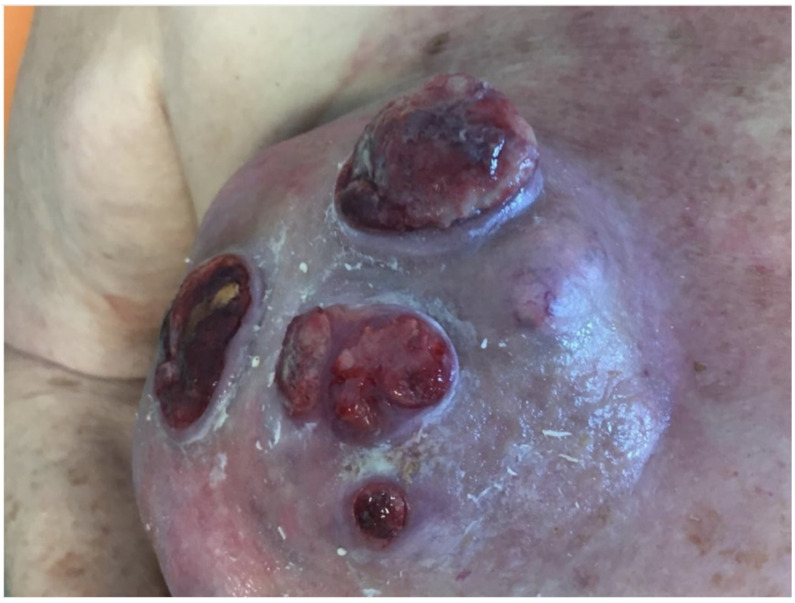
After seven days.

**Figure 4:**
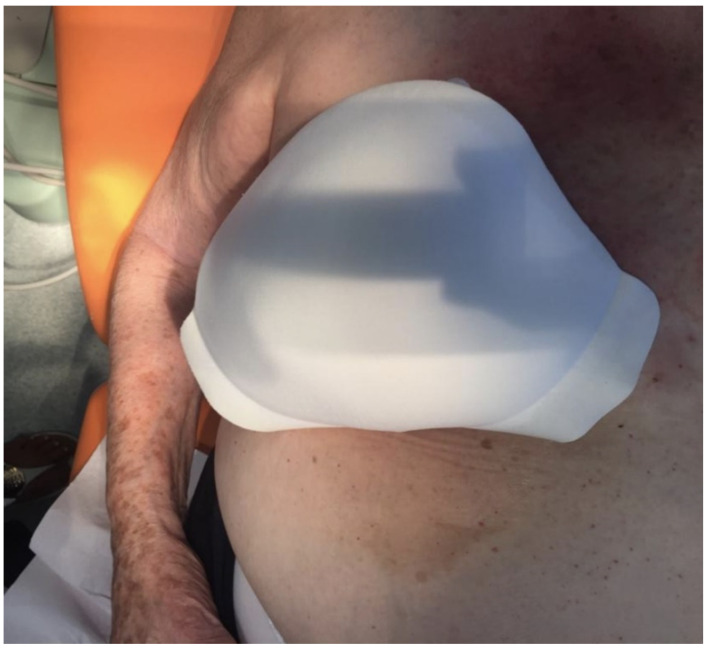
The gel device is applied again.

**Figure 5:**
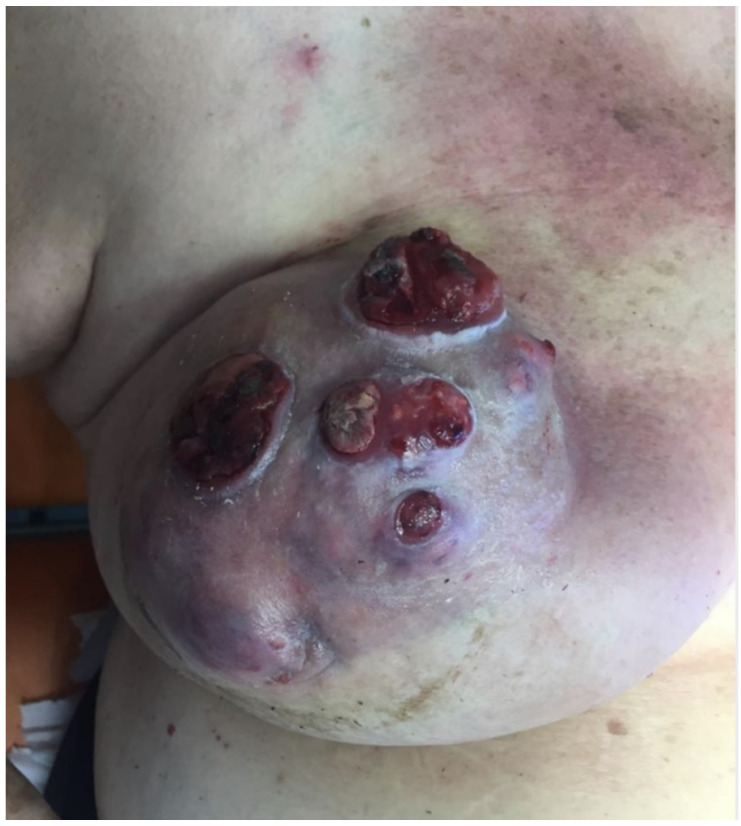
After 14 days.

**Figure 6:**
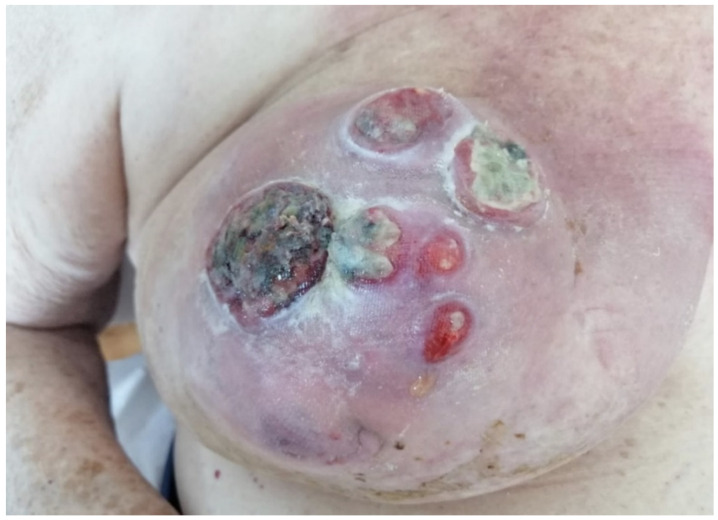
After 20 days.

**Figure 7:**
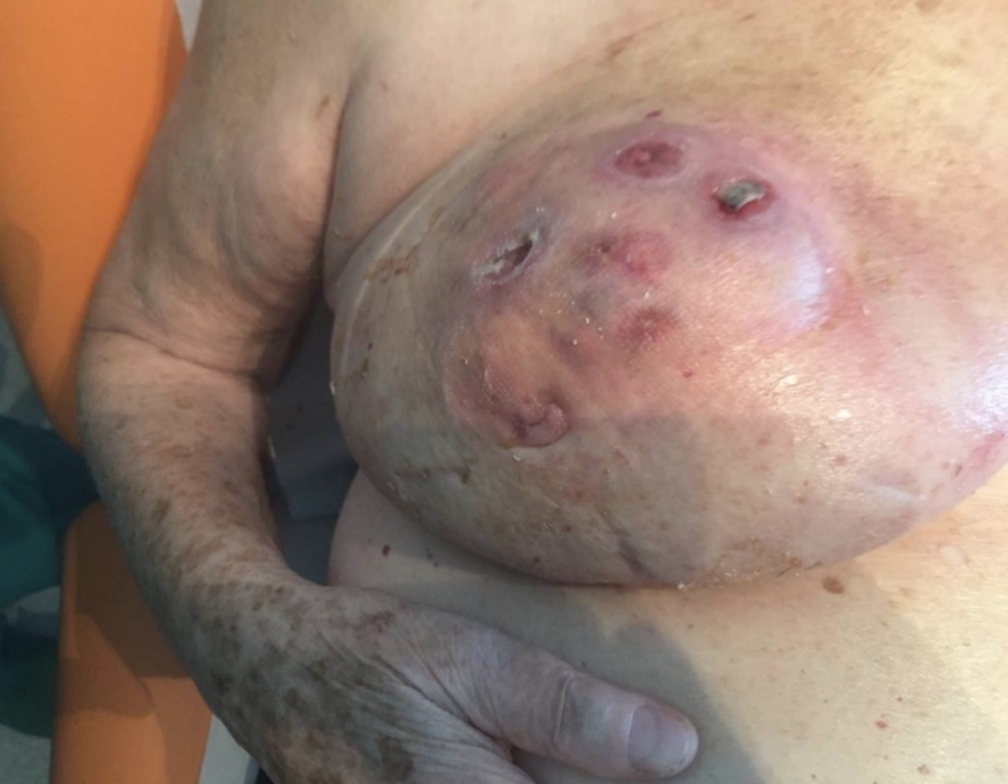
After 25 days.

**Figure 8:**
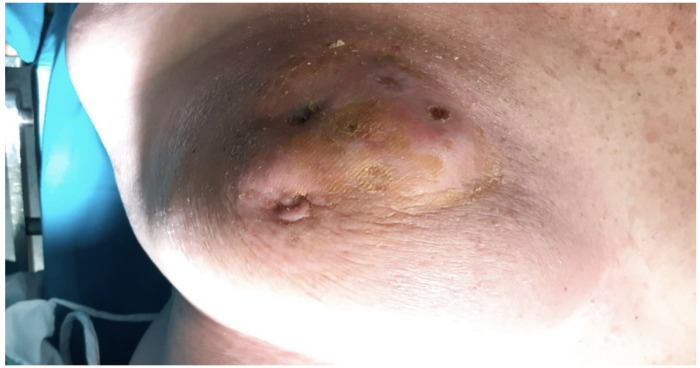
Picture after 30 days of application of the device. Clear local improvement of the skin and reduction of lesion areas.

The areas of necrosis, which was present in both groups at the beginning of the study, were significantly reduced after the first week of application of the device, together with changes in the perilesional skin. The reduced area was calculated to be 60% (see Figure 1, Figure 2, Figure 3, Figure 4, Figure 5, Figure 6, Figure 7 and Figure 8).

### 3.3. Impact on Quality of Life

The evaluation of the quality of life index shows a clear improvement in the treated group compared to the control. In the majority of cases, the patients reported a reduction in pain and exudate and the disappearance of the bad smell, which they all considered to be the main cause of isolation from people living with them.

## 4. Discussion

LABC represents an uncommon condition due to screening and surveillance programmes for breast lesions, and surgery is rarely a first-line option, since neoadjuvant therapies (chemotherapy and radiotherapy) act as a ‘bridge’ to surgery to remove the breast mass [5].

In the last decade the use of neoadjuvant (preoperative) chemotherapy has become the standard for large primary tumors or lymph node metastases [8,9,10,11].

National guidelines for breast cancer recommend that primary tumor surgery be reserved for patients with stage IV disease who have completed systemic therapy with “imminent complications, mainly local such as ulceration, bleeding and pain”.

Similarly, the treatment of LABC (locally advanced breast carcinoma) >5 cm in size with local tissue infiltration and skin ulceration, even in the absence of distant metastases, stage IIIB and IIIC, also requires a systemic approach in the first instance, particularly in cases of chronic lesions with peri-neoplastic inflammatory tissue, which constitute the framework of the so-called inflammatory carcinoma [5,8,10,12].

Ulcerated stage III and stage IV breast cancer remains difficult to treat representing both a clinical problem and a challenge in achieving local and distant control of the disease. Malignant wounds decrease the quality of life in 5% of patients with advanced cancers and 10% of patients with metastatic cancer. Often the average lifetime is estimated from 6 to 12 months. Pain is the most important aspect that reduces quality of life of these patients, especially in those cases with skin involvement. Nociceptors often stimulated persistent pain events at night and at rest, so a complete pain assessment is crucial for these patients in order to reduce local discomfort [13].

Chemotherapy resistance and poor tolerance to conventional treatments may result in poor tumor manageability, causing uncontrolled tumor growth, pain, bleeding and infections.

The often secreting, malodorous and painful lesions are a negative factor that severely affect patients’ quality of life and require daily medications that have little effect on inflammation and pain. In case of large and multiple lesions, malodorous exudate and pain may lead to depression and reduced social interaction [6].

In such a scenario, electrochemotherapy could be considered for selected patients as an alternative loco-regional treatment for disseminated cutaneous tumors or to improve patients’ quality of life as a palliative procedure [14].

To this purpose, Campana et al. published a study to evaluate the use of bleomycin associated with ECT employment on metastatic breast cancer in elderly patients [15].

The outcomes demonstrated ECT responsiveness in older and frail patients, achieving a rapid local tumor control. Overall the treatment was shown to be safe and tolerable, although adverse effects, such as pain and skin toxicity, may occur [15].

On the other hand, local treatment of wounds using medical devices catalogued as “advanced dressings” may interact with the wound bed by releasing antibacterial substances, reducing excessive exudate production, and thus preventing maceration of the wound edges or the possible formation of bacterial biofilm. However, these devices are usually used in case of non-neoplastic tissue and are therefore frequently inert in the microenvironment. Their use is limited to managing dressing changes, which are often painful, and to aiding repair processes that have been delayed by an event such as a chronic infection [12].

In the local treatment of neoplastic lesions, these advanced dressings have been used to allow daily management, e.g., containing exudate, reducing odour and stimulating proliferation of newly formed tissue. The main difficulty in patient management in this context is wound dressing because often the outpatient rooms are not provided with adequate advanced dressings.

In this regard, oxygen-enriched oil releasing reactive oxygen species (ROS) is considered an adjuvant to suppress bacterial colonization and pathogenesis proliferation, as well as a debriding agent promoting a microenvironment capable of stimulating angiogenesis in chronic and complicated wounds.

Reactive oxygen species (ROS), nitrogen species (RNS), carbon monoxide and hydrogen sulphite metabolites play an important autocrine and paracrine role in cellular signaling.

Various signals from the cellular microenvironment regulate the biosynthesis and catabolism of these mediators via mitochondria, nitric oxide synthase, heme oxygenase, oxidase, peroxidase, superoxide dismutase, cystathionine b-synthase and cystathionine c-lyase.

The activation of the synthesis and catabolism pathway of these signalling molecules is controlled by the transduction signalling pathway (activated by the binding of secreted factors to cell membrane receptors) and the mechanotransduction pathway (induced by changes in the rigidity of the extracellular matrix), which acts as an anchor for most cells. Matricellular proteins are a dynamically controlled subset of ECM proteins that play a key role in cellular redox signalling during development and in response to injury and stress. They differ from structural proteins, such as integrins, and appear transiently by binding to specific receptors through which they can perform intracellular signalling. These include thrombospondin-1, which regulates signalling for NO and H_2_S in the endothelium, thereby regulating tissue perfusion [16]. In general, it can be said that the redox system also intervenes directly or via the signaling function in the repair of the extracellular matrix in people with diseases such as diabetes [17,18,19].

ROS play a key role in biological processes and their production appears to be regulated by a mechanism between high and low intracellular concentrations. ROS dynamically influence the tumor microenvironment by promoting neoplastic angiogenesis, metastasis and cell survival at moderate concentrations through signalling with MAPK/ERK 1/2 (mitogen-activated proteinkinase/extracellular signal-regulated proteinkinase 1/2), p38, JNK (c-Jun N-terminal kinase) and P13K/Akt (phosphoinositide-3-kinase/protein kinase 3), which in turn activate NF-kB (nuclear factor kappa-light-chain-enhancer of activated B cells), metalloproteases (MMPs) and VEFG (vascular endothelial growth factor). At high concentrations, however, ROS can cause apoptosis of cancer cells. The causative conditions for these properties are not yet well defined, but it appears that tissue oxygenation conditions play an important role. The dual behaviour of ROS has directed research into possible anti-tumour applications [20]. In the literature, the application of reactive oxygen species, such as oxygen-enriched oils, has been evaluated in multiple disciplines and settings, including the treatment of chronic skin lesions, mainly due to their antiseptic properties, local reduction of inflammation and promotion of re-epithelization [2].

The concept of reactive species modulation (ROS) involves the release of constant quantities of reactive oxygen species for prolonged periods at low doses, differing substantially from other devices that incorporate large quantities of ozone, which, being highly unstable, tends to be immediately broken down into reactives and to exhaust its action in a short time.

ROS, including superoxide (O°), hydrogen peroxide (H_2_O_2_), free radicals and other reactive species are produced in cells as bioproducts of oxidative phosphorylation. ROS are capable of oxidative damage to lipids and proteins, which is why their levels are constantly controlled by scavenging enzymes and antioxidant molecules. Although they represent potential damage, signalling through these molecules is essential in tissue repair. Regulation of the redox system begins in the inflammatory phase when ROS reach their peak. Platelet aggregation in response to collagen damage induces the production of O- and 2 H_2_O_2_, which in turn amplify aggregation and recruit more platelets. ROS also contribute to the recruitment of inflammatory cells by stimulating chemotaxis and the expression of adhesion molecules [20,21].

High levels of O- and H_2_O_2_ are produced by neutrophils and macrophages via Nox. This ‘oxidative explosion’ is accompanied by a transient downregulation of scavenging enzymes. ROS also stimulate in this phase the release of cytokines and growth factors including colony-stimulating factor, platelet-derived growth factor (PFGF) and TNF-a. In the proliferative phase, ROS promote fibroblast proliferation and migration and mediate TGF-b1 signalling, resulting in collagen and fibronectin production and expression of basic fibroblast growth factor (bFGF). They also facilitate angiogenesis and H_2_O_2_ stimulates macrophage VEGF expression [21,22].

Although the mechanism by which neoplastic cells involve healthy tissues is still poorly understood, experimental research of both a biomolecular and genetic nature has clarified certain aspects of metabolism during the various phases of tumour growth. In recent decades, the disruption of the redox balance (oxidative stress) has been interpreted as one of the main causes of development, growth and metastasis in human cells. This imbalance is thought to be caused by an increase in free radicals, in particular ROS, the amount of which, in addition to being of intracellular origin, may be increased from outside [23].

ROS are normally produced in the mitochondria, endoplasmic reticulum and peroxisomes. Their quantity increases with the presence of inflammatory cells. Among ROS of external origin, environmental pro-oxidant toxins, radiation and various chemical compounds, including alcohol, smoking and some drugs, may play an important role. Free radicals are capable of damaging important molecules, such as lipids, proteins and nucleic acids, leading to oxidative stress in tissues [24].

High levels of ROS are accompanied by a downregulation of the antioxidant enzyme systems, which through various molecular pathways are capable of determining the formation of neoplasia. These pathways include NF-kB and the nuclear factor Nrf2 (erythroid derived2-like2 factor), which can generate signalling aimed at forming an inflammatory environment in which the tumour cell loses its capacity for apoptosis and tends towards proliferation and metastasis [25,26].

The study of the action of ROS in determining oxidative stress and the observation that oxygen levels in tissues tend to be low have focused attention on antioxidant substances, part of which are represented by enzymes such as superoxide dismutase (SOD), mitochondrial oxidative phosphorylation, p450 metabolism and peroxisomes, and the activation of inflammatory cells, such as macrophages and neutrophils. During mitochondrial respiration processes, 1–2% of oxygen is converted to ROS, which subsequently leads to the formation of hydroxyls, hydrogen peroxide, superoxides and free radicals [12,27,28].

The tumour environment thus creates a microenvironment with high levels of inflammatory factors, low oxygen levels and mitochondrial ROS production [28,29,30].

Biochemistry studies have revealed the role of oxidative phosphorylation and respiratory chains I and III in the mitochondrial level in determining an altered utilisation of pyruvate in the Krebs cycle. The availability of pyruvate for mitochondrial oxidation is regulated by pyruvate kinase (PKM), which catalyses the final stage of glycolysis into pyruvate. In cancer cells, there is up-regulation of the PKM2 isoform, which has reduced activity compared to the PKM1 isoform and allows the accumulation of intermediates that can be used for neoplastic tissue growth via the pentose phosphate pathway [29,30,31].

All forms of DNA damage, such as base modifications, chain breakage, cross-linking and protein production, are induced by ROS during the initial and growth phases of the neoplasm. Now, a paradox in biological systems is that ROS themselves can induce apoptosis of the neoplastic cell. ROS are capable of rupturing mitochondrial membranes and opening PTPs (permeability transition pores) and interfering with electron transfer chains by inducing the release of cytochrome c. In the cytosol, together with Apaf-1 (apoptopic peptidase activating factor 1) and procaspase 9, cytochrome c forms ‘apoptomes’ that can activate caspase 9 and other effectors with the result of triggering cell death. Hydrogen peroxide is one of the members of the ROS family that can directly induce apoptosis [17,19,21].

In our observational study, the local treatment of LABC-related skin lesions by the local application of an oxygen-enriched olive oil matrix gel favoured the improvement of the local clinical status.

The reduction of pain and the disappearance of the bad odour linked to the exudation of the lesions was favoured by the application of the polyester cup soaked in the above-mentioned compound.

In particular, Group A protocol was followed by a general improvement in local conditions with reduction in ulceration area, exudate and odour, while group B recorded approximately a 50% less reduction in the area of the lesions. We assessed all these aspects and registered a clear overall patient quality of life improvement in the treated group compared to the control group.

The application of the device did not lead to any side effects or complications requiring interruption of treatment. In some cases, the patients reported a transient and bearable burning sensation that disappeared a few hours after application.

From the condition of the perilesional skin to the characteristics of the neoplastic tissue that gradually reduces the signs of inflammation, it is evident that the application of the oleic matrix allows a consistent aid to neoadjuvant therapy by keeping the lesion clean and improving the oxygenation conditions of the edges. In the case reported in the photographic diary it is possible to clearly observe the changes that occurred locally on the lesions after the first applications of the device, which indicate that the clinical conditions have improved considerably, preparing the field for a safer and favorable surgical approach. A limit to this study is represented by the small cohorts considered in the two groups. In fact, the scarcity of cases of severe local advanced breast cancers did not allow the authors to enroll a larger number of patients. However, we believe this innovative paper sets the basis for further investigations in this evolving field.

## 5. Conclusions

The results obtained in this preliminary study, although numerically and statistically insignificant, suggests that the application of the abovementioned devices in the setting of LABC skin lesions with ulcerated and neoplastic tissue, represents a safe and effective alternative to traditional dressings and does not lead to the onset of negative effects due to the release of ROS. The key role of oxygen-enriched oil releasing reactive oxygen species (ROS) in tissue repair requires further studies to fully understand their potential and expand the fields of application of the device by exploiting its modulation capabilities.
